# An Update of the Cenchrinae (Poaceae, Panicoideae, Paniceae) and a New Genus for the Subtribe to Clarify the Dubious Position of a Species of *Panicum* L.

**DOI:** 10.3390/plants12040749

**Published:** 2023-02-07

**Authors:** Carolina Delfini, Sandra S. Aliscioni, Juan M. Acosta, José F. Pensiero, Fernando O. Zuloaga

**Affiliations:** 1 Instituto de Botánica Darwinion (ANCEFN–CONICET), Labardén 200, Casilla de Correo 22, San Isidro B1642HYD, Buenos Aires, Argentina; 2 Cátedra de Botánica General, Facultad de Agronomía, Universidad de Buenos Aires, Av. San Martín 4453, Buenos Aires C1417DSE, Argentina; 3 Instituto de Ciencias Agropecuarias del Litoral, UNL–CONICET–FCA, Kreder 2805, Esperanza 3080HOF, Santa Fe, Argentina

**Keywords:** bristle clade, *Janochloa*, *ndh*F, *Panicum antidotale*, *Paspalidium*, *Paurochaetium*, *Reverchoniae*

## Abstract

Subtribe Cenchrinae, so-called as the “bristle clade”, is a monophyletic group of panicoid grasses characterized by having sterile branches or bristles on the inflorescences in most of its species. Within this subtribe is also placed *Panicum antidotale* Retz., an “incertae sedis” species of *Panicum* L. which lacks bristles along the inflorescence. In this study, we present an update of the subtribe Cenchrinae based on molecular, morphological, and anatomical evidence to clarify the systematic position of *P. antidotale* in the Cenchrinae, excluding it from *Panicum* and establishing it in a new genus (i.e., *Janochloa* Zuloaga & Delfini); the morphological features distinguishing the new genus from other closely related taxa are properly discussed and an identification key to the 24 genera recognized within Cenchrinae is presented. We also add American *Setaria* species, not tested before, of subgenera *Paurochaetium* and *Reverchoniae*, discussing the position of these taxa in actual phylogeny of the genus as well as defining placements in the tree of *Setaria* species that were imprecisely located in previous analyses. A comparison with the results from other studies, comments on *Stenotaphrum* Trin. and a brief discussion on conflicting placements in *Cenchrus* and related taxa, and of *Acritochaete* Pilg. are also included.

## 1. Introduction

Subtribe Cenchrinae Dumort., of the Paniceae R. Br., consists of 24 genera morphologically defined by the presence of sterile branches or bristles on the inflorescence in the majority of the species; this subtribe is a sister to subtribe Melinidinae, and both form a monophyletic group with subtribe Panicinae that is referred to as the MPC clade, well-supported in diverse phylogenies based on chloroplast, mitochondrial, and nuclear rDNA gene sequence [1,2,3,4,5]. Species of the Cenchrinae are also homogeneous, in terms of photosynthesis, because all genera and species are of the C_4_ NADP-me subtype (see comments on *Acritochaete* Pilg. below); on the other hand, taxa of subtribe Melinidinae are of the PCK subtype, while all Panicinae are of the NAD-me subtype [6,7,8]. Although monophyly of subtribe Cenchrinae was consistent in all phylogenetic studies [1,5,9], resolution of basal clades is low; therefore, with the relationship of genera, in many cases, still doubtful.

Most genera of the subtribe have in common bristles, from one to many, and persistent to deciduous, on the inflorescence. However, there are four genera that lack bristles along the inflorescence, i.e., 1. *Whiteochloa* C.E. Hubb., with six species in Australia and the Pacific Islands; the genus was originally classified in the Panicinae [2] but later confirmed in the Cenchrinae [10]; 2. *Alexfloydia* B.K. Simon, with one species restricted to southeastern Australia; 3. *Zuloagaea* Bess, monotypic and segregated from *Panicum* L. (*P. bulbosum* Kunth); and 4. *Panicum antidotale* Retz., also included in the Cenchrinae, which is a still incertae sedis species of *Panicum*, originally placed in an unassigned section of subgenus *Agrostoidea* [11]. The phylogenetic placement of *P. antidotale*, within the Cenchrinae, and its relationship with other genera of the subtribe is still doubtful and in need of a systematic definition. Another genus of doubtful position, within the Cenchrinae, is *Acritochaete*, the single C_3_ taxon remaining in the subtribe.

Among the genera with bristles in the inflorescence, the Cenchrinae comprises two large and very diverse genera: *Cenchrus* L. and *Setaria* P. Beauv. Species of *Cenchrus* are in a strong clade; the genus was expanded to include species of *Cenchropsis* Nash, *Kikuyuochloa* H. Scholz, *Odontelytrum* Hack., *Pennisetum* Rich. and *Snowdenia* C.E. Hubb. [1,12,13,14]. Species of *Cenchrus* s.l. have inflorescences with an abscission zone at the base of the primary branch, i.e., spikelets fall surrounded by an involucre of bristles [15]. *Setaria*, as presently circumscribed, is a cosmopolitan genus of grasses comprising between 115 and 160 species [16,17], commonly found in open environments and woodlands [17,18]. The species grow mostly in tropical and subtropical latitudes, though several are present in cold regions of both hemispheres [17,18]. The Old World species are concentrated in tropical Africa, including 12 endemic to Madagascar, whereas in the New World the center of species diversity is Brazil with 30 native species [19,20,21,22,23]. Opposite to *Cenchrus*, *Setaria* is one of several genera in Cenchrinae that the setae or bristles persist when the spikelets fall at maturity [14,16,17]. Regarding the infrageneric classification of *Setaria*, in general, one or two distinctive groups of species have been recognized, and the remainder are placed in subgenus/section *Setaria* [18,20,24]. For tropical African species, [19] there are four recognized sections, that is, *Eu-Setaria* Stapf, characterized by its young blades, not plicate, and panicles usually spikelike; *Ptychophyllum* (A. Braun) Stapf, including plants with plicate blades and open panicles; and sections *Panicatrix* Stapf & C.E. Hubb. and *Cymbosetaria* Stapf & C.E. Hubb., distinguished by having rounded and keeled upper lemmas, respectively, and non-cylindrical inflorescences. In addition to these four, [24] section *Setaria* is also recognised, characterized by the blades not plicate and bristles usually below all the spikelets. Using similar criteria and also based on the position of the bristles along the inflorescences, [20] recognized three subgenera for the North American species: *Setaria*, *Ptychophyllum* and *Paurochaetium* (Hitchc. & Chase) Rominger, which group species with bristles present only at the ends of primary branches. [18], in his treatment of the South American species, recognized the subgenera *Setaria* and *Ptychophyllum* and proposed the new monotypic subgenus *Cernatum* Pensiero to accommodate *Setaria cernua* Kunth, whose position in the infrageneric classification had long been uncertain.

In some species of *Setaria*, the primary branches of the inflorescence are themselves unbranched (i.e., the spikelets are born directly on the primary branches) and these branches end in a bristle [17]. The Old World species with this type of inflorescences, called informally “Paspalidium type”, were placed in the genus *Paspalidium* Stapf [25]. However, American species with similar type of inflorescences were treated in three different subgenera within *Setaria*: *Paurochaetium*, *Reverchoniae* W.E. Fox (segregated from subgenus *Paurochaetium*), and *Cernatum* [18,20,26]. Advances in morphological and systematic studies in *Setaria* have shown that there is another type of inflorescence in which some other spikelets (but not all) are associated with bristles in addition to the uppermost ones in the branch tip, although the general pattern is similar to the “Paspalidium type” [15,17]. The recognition of this intermediate pattern led to the transfer of Old World *Paspalidium* species back to *Setaria* [16,17,27,28], a result partially supported by molecular phylogenies [1,14,29,30], and morphological and foliar anatomical data [31].

The most complete phylogeny of *Setaria* and its related genera lacks the resolution of the backbone of the tree [15,29]. It is based on the plastid marker *ndh*F and shows the genus to be para- or polyphyletic, with a number of well-supported clades corresponding largely to geography [15,29]. The absence of a well-resolved phylogeny along with the difficult morphological delimitation of some species means that *Setaria* requires further in-depth research. It is clearly not a natural group, but more evidence is still needed to allow restructuring of the taxonomy of *Setaria*. For this reason, our purpose here is to give more information about species of the genus, and discuss the results, without pursuing a solution to its intricate phylogeny.

The aim of this study was to increase the knowledge of taxa of the Cenchrinae through: (a) solving the systematic position of *Panicum antidotale* in the Cenchrinae, excluding the species from *Panicum* and establishing a new genus; (b) adding American *Setaria* species, not tested before, of subgenera *Paurochaetium* and *Reverchoniae*, discussing the position of these taxa in actual phylogeny of the genus; (c) analysing Old World species of *Setaria* and other genera of Cenchrinae from Genbank not considered in previous phylogenies [29]; and (d) presenting new accessions of some other New World species of *Setaria* (not *Paurochaetium* and *Reverchoniae*), in order to confirm their placement in the phylogenetic tree, and more vouchers of some species that were imprecisely located in [29], as they were represented by partial, not fully double-stranded and/or poor-quality accessions. In addition, we are including a new key to recognize all genera of the Cenchrinae.

## 2. Results

**Figure 1:**
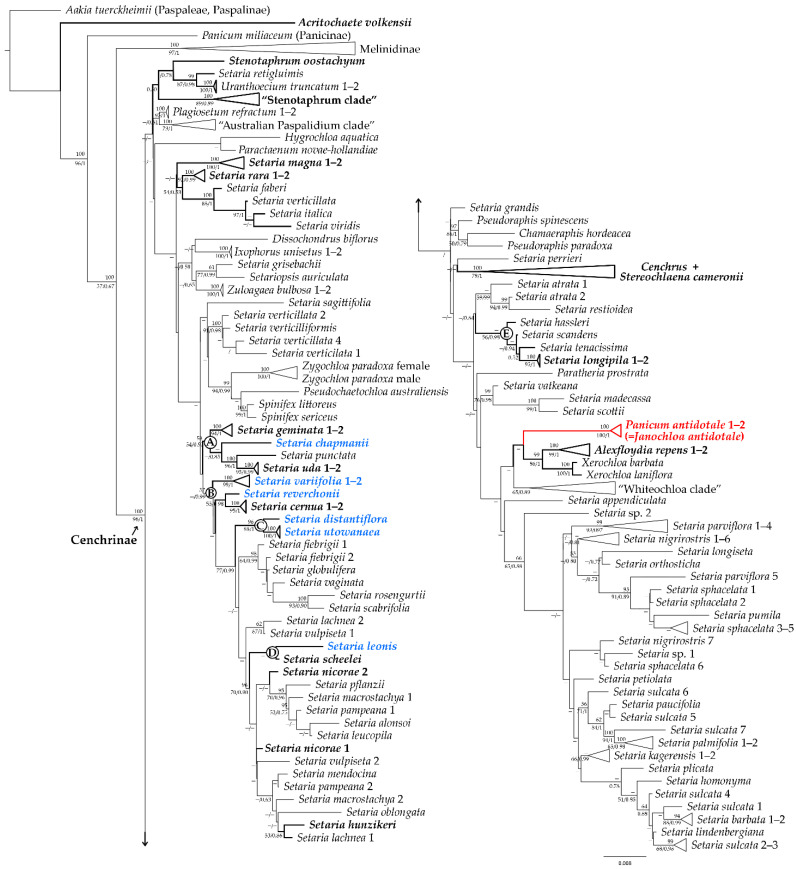
Maximum Clade Credibility Tree (MCCT) from the Bayesian inference analysis based on the chloroplast *ndh*F gene. Bootstrap supports from parsimony are listed above the branches and bootstrap supports from maximum likelihood/posterior probabilities from Bayesian inference are listed below the branches. Nodes with “–” have posterior probabilities and bootstrap supports <0.50 and <50%, respectively. Clades/taxa in bold and/or denoted by letters are discussed in the text. The new genus, *Janochloa* Zuloaga & Delfini, is highlighted in red, and the species of subgenera *Paurochaetium* and *Reverchoniae* of *Setaria* in blue.

The aligned data matrix for 180 accessions consists of 2077 nucleotide positions, of which 278 characters were phylogenetically informative. The parsimony analyses found 80 trees of 776 steps (uninformative characters excluded), with a consistency index (CI) of 0.466 and a retention index (RI) of 0.804. The strict consensus tree from MP, the Maximum Clade Credibility Tree (MCCT) from BI and the ML tree produced similar topologies showing the same strongly supported clades; thus, only the MCCT from BI is presented here along with branch supports obtained under MP and ML analyses (Figure 1). The aligned data matrix and trees from the three methods of analysis are available at Repositorio Institucional CONICET Digital under the following link: http://hdl.handle.net/11336/163438 (accessed on 28 July 2022).

For eight taxa, we were able to sequence a second voucher (i.e., *Alexfloydia repens* B.K. Simon, *Panicum antidotale*, *Setaria cernua* Kunth, *Setaria geminata* (Forssk.) Veldkamp, *Setaria magna* Griseb., *Setaria nicorae* Pensiero, *Setaria rara* (R. Br.) R.D. Webster, and *Setaria uda* (S.T. Blake) R.D. Webster, as well as two accessions for *Setaria longipila* E. Fourn., *Setaria utowanaea* (Scribn.) Pilg., and *Setaria variifolia* (Swallen) Davidse [see Table S1 available in Supplementary Material here]. Except for *S. nicorae*, whose mutations in the sequences led its accessions to distinct placements (i.e., separated into two clades), the two accessions of the same species had identical or nearly identical sequences and were placed together by the three analyses (Figure 1).

Based on the phylogenetic evidence presented here, the subtribe Cenchrinae, as currently circumscribed, is paraphyletic since *Acritochaete volkensii* Pilg. was placed outside the “bristle clade”, unrelated to the remaining genera of the subtribe. Although the two accessions of *Panicum antidotale* were consistently placed together (Bayesian posterior probability (BPP) 1/ML bootstrap (MLB) 100/parsimony bootstrap (PB) 100) embedded in the Cenchrinae, its position in the tree is unclear. In the Bayesian analysis *Panicum antidotale* formed a weakly supported clade (BPP < 0.50) with the two accessions of *Alexfloydia repens*, *Xerochloa barbata* R. Br., and *Xerochloa laniflora* Benth. (Figure 1), while the two bootstrap analyses recovered it as a sister to *Paratheria prostrata* Griseb, a relationship also with almost no support (MLB and PB < 50).

Our phylogeny showed the *Setaria* species/accessions analyzed distributed in different clades through the tree (A–E), being *Setaria magna* Griseb. and *Setaria rara* (R. Br.) R.D. Webster in an uncertain position. Species of subgenera *Paurochaetium* and *Reverchoniae* (see Supporting Information Table S2) were placed into two clades and two subclades (A–D; Figure 1), as detailed next.

Clade A is a strongly supported clade in BI (BPP 0.93/MLB 54/PB 59), including the two accessions of *S. uda* (BPP 0.99/MLB 95/PB 100) as sisters to *Setaria punctata* (Burm. f.) Veldkamp (BPP 1/MLB 96/PB 100), and both related to *Setaria chapmanii* (Vasey) Pilg. (BPP 0.85/MLB < 50/PB < 50); the two accessions of *Setaria geminata* (Forssk.) Veldkamp (BPP 1/MLB 94/PB 100) are strongly (BI) supported as sister group to all clade A species. Clade A was also resolved in a weakly (BI) supported position sister to clade B, which groups most of the *Setaria* species from the Americas. The remainder species of the subgenera *Paurochaetium* and *Reverchoniae* (i.e., *Setaria distantiflora* (A. Rich.) Pilg., *Setaria leonis* (Ekman ex Hitchc.) León, *Setaria reverchonii* (Vasey) Pilg., *S. utowanaea*, and *S. variifolia*; Table S2) were placed within clade B but they do not appear to be related to each other, except for the two accessions of *S. utowanaea* (BPP 1/MLB 100/PB 100), strongly related to *S. distantiflora* in subclade C (BPP 1/MLB 98/PB 96). The two accessions of *S. variifolia* were consistently placed together (BPP 1/MLB 99/PB 100) and supported as a sister group to all clade B species. Within subclade D, the two accessions of *Setaria cernua* Kunth (BPP 1/MLB 95/PB 100) are closely related to *S. reverchonii* with a strong branch support in the BI (BPP 0.98/MLB 55/PB < 50). Bayesian analysis also found weak support (BPP < 0.50) for a sister relationship between *S. leonis* and *S. scheelei*, but this grouping was not supported in ML and MP.

The two accessions of *Setaria nicorae* Pensiero are also included within the larger American clade (clade B), but unrelated to each other. *Setaria hunzikeri* Anton is nested with one accession of *Setaria lachnea* (Nees) Kunth in Bayesian and ML analyses (BPP 0.86/MLB < 50) and not recovered in MP.

The American *Setaria* species of clade E, strongly supported in the BI analysis (BPP 0.98/MLB 56/PB < 50,), are not related to the members of the larger American clade. It includes the two accessions of *Setaria longipila* E. Fourn. placed together (BPP 1/MLB 96/PB 100) with *Setaria tenacissima* Schrad., *Setaria scandens* Schrad., and *Setaria hassleri* Hack. as its successive sisters (BPP 0.52/PB < 50; and BPP 0.94/MLB < 50/PB < 50, respectively).

*Setaria magna* and *S. rara* are the only two *Setaria* species that have not been consistently assigned/related to any of the retrieved clades. Although their two accessions were unambiguously supported together (BPP 1/MLB 100/PB 100; BPP 0.99/MLB 92/PB 100, respectively), their placements in this phylogeny remain unclear. Both species were poorly BI and ML supported as successive sisters to four temperate Asian species (i.e., *Setaria faberi* R.A.W. Herrm., *Setaria italica* (L.) P. Beauv., *Setaria verticillata* (L.) P. Beauv. (3), and *Setaria viridis* (L.). P. Beauv.) (BPP 0.53/MLB < 50), but MP did not retrieve this relationship.

*Cenchrus* and *Stenotaphrum* Trin. were resolved as paraphyletic genera. *Stereochlaena cameronii* (Stapf) Pilg. was included in the “Cenchrus clade” (BPP 1/MLB 79/PB 100), while *Stenotaphrum oostachyum* Baker was placed outside the “Stenotaphrum clade” (BPP 0.99/MLB 89/PB 100), related to *Setaria retiglumis* (Domin) R.D. Webster and *Uranthoecium truncatum* (Maiden & Betche) Stapf (BPP 0.78/MLB < 50/PB < 50).

## 3. Discussion

### 3.1. Monophyly of the Cenchrinae

The monotypic *Acritochaete volkensii* Pilg. is an annual species found in shady forests of tropical Africa [32,33], currently treated within the subtribe Cenchrinae [14]. It has inflorescences bearing persistent setae, a character state shared with the “bristle clade”; however, our results placed *A. volkensii* outside the Cenchrinae, as also indicated in [3,5]. Despite the morphological similarities with Cenchrinae, the photosynthetic C_3_ pathway of *A. volkensii* is absent within this subtribe, which includes only C_4_ NADP-me plants [1,14]. According to [3], *Acritochaete* Pilg. is closely related to members of the Boivinellinae Pilg. subtribe known as “the forest shade clade” [3,34], and which groups mostly physiologically C_3_ genera [1,14]. Thus, for both subtribes to be monophyletic, *A. volkensii* must be recognized within the subtribe Boivinellinae.

### 3.2. Panicum antidotale Retz., an Odd Species in the Subtribe Cenchrinae

Polyphyly of the genus *Panicum* has been confirmed in several morphological and phylogenetic studies of Panicoideae based on chloroplast (*ndh*F, *trn*L-F, *rpo*C2) and ribosomal nuclear (ETS) sequence data [10,34,35,36,37,38,39]. Based on four plastid markers, [40,41] also indicated that *Panicum* should be restricted to a set of species all using the C_4_ NAD-me photosynthesis subtype. [10] considered *Panicum* s. str. as a genus with nearly 163 worldwide distributed, and characterized as including caespitose plants, with ciliate or membranous-ciliate ligules, spikelets arranged in an open and lax inflorescence, with the upper glume and lower lemma (5–)7–13-nerved, the upper anthecium indurate, and the upper palea with simple or compound papillae toward the apex. Species of the genus were classified in subtribe Panicinae of Paniceae, together with the genus *Louisiella* C.E. Hubb. and J. Léonard., a pantropical genus with two species. As a result of these phylogenetic studies, species of *Panicum* s.l. were transferred to other panicoid genera such as *Dichanthelium* (Hitchc. & Chase) Gould, *Hymenachne* P. Beauv., *Steinchisma* Raf., and *Phanopyrum* (Raf.) Nash, or classified in new genera, either monotypic or with up to nearly 40 species such as *Aakia* J.R. Grande [42], *Adenochloa* Zuloaga [43], *Apochloa* Zuloaga & Morrone [44], *Canastra* Morrone, Zuloaga, Davidse & Filg. [45], *Cnidochloa* Zuloaga [46], *Coleataenia* Griseb. [47], *Cyphonanthus* Zuloaga & Morrone [48], *Hopia* Zuloaga and Morrone [49], *Morronea* Zuloaga & Scataglini [50], *Ocellochloa* Zuloaga & Morrone [51], *Parodiophyllochloa* Zuloaga & Morrone [52], *Renvoizea* Zuloaga & Morrone [44], *Rugoloa* Zuloaga [53], *Stephostachys* Zuloaga & Morrone [54], *Trichanthecium* Zuloaga & Morrone [55], and *Zuloagaea* Bess [56]. All previouly mentioned genera were classified under three different subtribes of tribe Paspaleae (Arthropogoninae, Paspalinae, and Otachyriinae) and five subtribes of Paniceae: Boivinellinae, Cenchrinae, Dichanthelliinae, Melinidinae, and Panicinae.

Although these studies represent an important advancement in the evolutionary knowledge of the Panicoideae, there are still “incertae sedis” species of *Panicum* in need of a taxonomic definition. Among these taxa, [14] included 18 non Kranz species in the “*Sacciolepis* grex”, 12 non Kranz species in the Boivinellinae, 3 Kranz PEP-ck species in the Melinidinae, and one Kranz NADP-me species in the Cenchrinae. The single “incertae sedis” species of *Panicum* within the Cenchrinae is *P. antidotale*, introduced all over the world due to its medicinal, environmental and animal food uses, being the native range from Sinai to Indo-China [33]. The species is characterized by having a perennial habit with strong rhizomes, rigid and solid internodes, lax panicles, glabrous spikelets, the lower glume ½ or more the length of the spikelet, and upper glume and lower lemma 5–9-nerved [21]. As previously stated, *P. antidotale* is the only remaining C_4_ “*Panicum*” species of the MS type (XyMS–) that has a carbon isotope value of −14.1 and which anatomically has a single mestome Kranz sheath around the vascular bundles, centrifugal arrangement of chloroplasts, and three mesophyll cells between vascular bundles [57,58]; these characteristics obviously imply that the species must be segregated from *Panicum* s. str.

The placement of *P. antidotale* in the “bristle clade” was first indicated by [39] basing on the chloroplast *ndh*F data and using a sequence obtained from a single correctly identified accession. By the addition of a second voucher specimen and both accessions consistently placed together, our data confirmed that *P. antidotale* is related to members of subtribe Cenchrinae, although its position in the tree is unresolved.

As in other three genera of Cenchrinae (i.e., *Alexfloydia* B.K. Simon, *Whiteochloa* C.E. Hubb. and *Zuloagaea*), *P. antidotale* has spikelets and inflorescences morphologically similar to those of *Panicum* s. str. and lacks setae [1]. *Alexfloydia*, a monotypic genus from Australia, is a stoloniferous perennial, with decumbent culms, branching at the lower nodes, with ciliate ligules, terminal inflorescences, and few flowered, spikelets laterally compressed. Spikelets are adaxial; the lower glume is 5–7-nerved, ¾ the length of the spikelet; the upper glume and lower lemma are 9-nerved, the lower flower is male, and the upper anthecium is cartilaginous and smooth [59]. *Whiteochloa* C.E. Hubb., with six species distributed in Australia, New Guinea, and neighboring islands, includes annual or perennial plants, with shortly rhizomatous perennial; its internodes are hollow, its ligules membranous-ciliate to ciliate, and its inflorescence a panicle composed of unilateral racemes, laterally-compressed spikelets. The lower glume is 3–5-nerved, and the upper glume and lower lemma are 5–7-nerved; the lower flower is male, and the upper anthecium is stipitate, indurate and rugose [60]. *Zuloagaea* is also a monotypic genus growing from United States to northern South America, Colombia to Ecuador, characterized by its corm-like base, membranous ligules, open and pyramidal inflorescences, dorsiventrally compressed spikelets, 3-nerved, lower glume, ⅔ the length of the spikelet, the upper glume and lower lemma are subequal and 5-nerved; the lower palea is hyaline, the lower flower is male or absent, and the upper anthecium is not stipitate; rugose and indurate [56].

Despite its divergent morphology, *P. antidotale* shares with the remainder Cenchrinae a Kranz leaf blade anatomy typical of the NADP-me subtype photosynthetic pathway [10,57,58]. Ref. [56] hypothesized the possibility that *Zuloagaea bulbosa* has cryptic setae that would link it to the more closely related species of *Setaria* and *Ixophorus*, which, perhaps, could also have occurred in *P. antidotale*, *Alexfloydia repens*, and in *Whiteochloa*; developmental patterns of inflorescences in these species should be studied to determine the basis for the absence of bristles [1].

Based on previous studies [1,14,30,39] and the evidence presented here, we believe that “*Panicum antidotale*” needs to be treated as a monospecific genus within the Cenchrinae in order to take into account the evolutionary history of the subtribe as currently known and to provide a means of reference to facilitate communication about these taxa. Monotypic taxa are a challenging group that deserve attention [61]. Even when we acknowledge that the erection of monotypic genera is a controversial matter (e.g., [62,63]), here we propose to recognize one new monotypic genus in Paniceae, subtribe Cenchrinae, a decision supported by both morphological and molecular data. The erection of *Janochloa* Zuloaga & Delfini increase the total number of genera in the Cenchrinae to 24, once *Acritochaete* is excluded (see previous comments on item 3.1). This subtribe comprehends 12 monotypic genera, seven of them endemic to Australia (i.e., *Alexfloydia*, *Chamaeraphis* R. Br., *Hygrochloa* Lazarides, *Paractaenum* P. Beauv., *Plagiosetum* Benth., *Pseudochaetochloa* Hitchc., *Uranthoecium* Stapf, and *Zygochloa* S.T. Blake), two endemic to America, from the United States, Mesoamerica and the Caribbean to northern South America (i.e., *Ixophorus* Schltdl., and *Zuloagaea*), one endemic to Africa (*Streptolophus* Hughes), and the last one, *Dissochondrus* (Hillebr.) Kuntze, endemic to Hawaii. A total of eight genera are oligotypic and have a narrow distribution with up to eight species: two of them, *Whiteochloa*, 6 spp., and *Xerochloa* R. Br., 3 spp., restricted to Australasia, *Pseudoraphis* Griff., 8 spp., and *Spinifex* L., 4 spp., growing in Asia and Australasia, *Stereochlaena* Hack., 4 spp., present only in Africa, *Setariopsis* Scribn. ex Millsp., 2 spp., endemic from the United States to Colombia and Venezuela; *Holcolemma* Stapf and C.E. Hubb. has 3 species in Africa, Asia, and Australasia, and *Paratheria* Griseb., 2 spp., is present in Africa, Madagascar, and America. Finally, three genera have a cosmopolitan distribution: *Stenotaphrum* Trin (7 spp.), *Cenchrus*, with 120 species, and *Setaria*, with nearly 115–150 species.


**Key to Genera of the Subtribe Cenchrinae:**
1. Inflorescence subtended by a spatheole…………………………………………………….2 1′. Inflorescence without a spatheole below…………………………………………………..4 2(1). Plants bisexual…………………………………………………………………..***Xerochloa*** 2′. Plants dioecious……………………………………………………………………………….3 3. Male spikelets different from female ones; male inflorescence a single raceme…………………………………………………………………………….……....***Spinifex*** 3′. Male spikelets similar to the female ones; male inflorescence similar to the female one……………………………………………………………………………………...***Zygochloa*** 4(1′). Inflorescence composed of short racemes with spikelets embedded in a thickened central axis…………………………………………………………………………………***Stenotaphrum*** 4′. Inflorescence without spikelets embedded in a thickened central axis…………………5 5(4′). Inflorescence composed of paired or digitate racemes…………………***Stereochlaena*** 5′. Inflorescence open to contracted or spiciform, not composed of paired or digitate racemes……………………………………………………………………………………………6 6(5′). Spikelets with two fertile florets. Endemic of Hawaii…………………***Dissochondrus*** 6′. Spikelets with a male, or neuter, floret below and a fertile floret apically (or male and female spikelets in different inflorescences)…………………………………………………..7 7(6′). Species with sexes segregated on unisexual branches; male spikelets above and female spikelets below………………………………………………………………………………....***Hygrochloa*** 7′. Species without sexes segregated on unisexual branches………………………………...8 8(7′). Caryopsis with a linear hilum……………………………………………..***Pseudoraphis*** 8′. Caryopsis with a punctiform to oblong hilum…………………………………………….9 9(8′). Inflorescence with disarticulation at maturity at the base of the primary branches or at the nodes of the inflorescence axis………………………………………………………………………………………………..10 9′. Inflorescence not disarticulating at the base of the primary branches…………………15 10(9). Lower palea winged, expanded at maturity. Africa…………………...***Uranthoecium*** 10′. Lower palea not expanded at maturity. Australia……………………………………...11 11(10′). Lower glume developed, 3–7-nerved; upper glume ovate, 11–19-nerved (except ***Pseudochaetochloa***, with upper glume 3-nerved and plants perennial); plants annual…...12 11′. Lower glume absent or obscure, nerveless; upper glume 0–7-nerved; plants perennial…………………………………………………………………………………………14 12(11). Upper glume 3-nerved; plants perennial………………………..***Pseudochaetochloa*** 12′. Upper glume 11–19-nerved; plants annual……………………………………………...13 13(12′). Spikelets surrounded by a fan of bristles……………………………….***Plagiosetum*** 13′. Bristle single…………………………………………………………………...***Paractaenum*** 14(11′). Inflorescences of racemes in a bilateral false spike; lower glume linear, almost as long as the lower lemma, 7-nerved. Australia………………………………………………………………………..…***Chamaeraphis*** 14′. Inflorescences of racemes in a multilateral false spike; lower glume reduce; nerveless. Africa and America………………………………………………………………………………..***Paratheria*** 15(9′). Inflorescences without bristles………………………………………………………...16 15′. Inflorescences subtended with 1 to many bristles or spines…………………………...19 16(15). Spikelets laterally compressed………………………………………………………...............................................17 16′. Spikelets dorsiventrally compressed……………………………………………………..18 17(16). Ligule membranous-ciliate; inflorescence composed of unilateral racemes; upper glume and lower lemma 5–7-nerved; upper anthecium stipitate, rugose, and coriaceous…………………………………………………………………………...***Whiteochloa*** 17′. Ligule ciliate; inflorescence with spikelets arranged in open, not racemose branches; upper glume and lower lemma 7–9-nerved; upper anthecium not stipitate, smooth, cartilaginous………………………………………………………………………….***Alexfloydia*** 18(16′). Plants with the lowest internodes developed into a corm-like base, without strong rhizomes; internodes hollow; ligules membranous; upper anthecium rugose…………………………………………………………………………………..***Zuloagaea*** 18′. Plants without corm-like bases, strongly rhizomatous; internodes solid; ligules membranous-ciliate; upper anthecium smooth…………………………………….***Janochloa*** 19(15′). Bristles persistent on the inflorescence………………………………………………20 19′. Bristles or spines falling with the spikelets…………………………………………...…21 20(19). Lower palea expanded at maturity; C_3_ genus…………………………..***Holcolemma*** 20′. Lower palea not expanded at maturity; C_4_ genus………………………………...***Setaria*** 21(19′). Spikelet subtended by a single bristle………………………………………………22 21′. Spikelet surrounded by an involucre of bristles………………………………………...23 22(21). Panicle contracted, spiciform, first order branches appressed, short; lower palea not expanded at maturity…………………………………………………………………………….…***Setariopsis*** 22′. Panicle open, first order branches divergent, long; lower palea expanded and winged at maturity………………………………………………………………………………...***Ixophorus*** 23(21′). Inflorescence with distant racemes-like branches; leaf blades pseudopetiolate…………………………………………………………………...***Streptolophus*** 23′. Inflorescence spiciform; leaf blades not pseudopetiolate……………………...***Cenchrus***

#### Taxonomic Treatment

***Janochloa*** Zuloaga & Delfini, **gen. nov.** TYPE SPECIES: ***Janochloa antidotale*** (Retz.) Zuloaga & Delfini.

Long-rhizomatous perennial, with decumbent culms and solid, glaucous internodes. Ligules membranous-ciliate; blades lanceolate. Inflorescence a lax and a diffuse panicle. Spikelets long ovoid, chasmogamous and glabrous. Lower glume ¾ as long as the spikelet, 3–5-nerved. Upper glume and lower lemma subequal and 5–9-nerved. Lower palea elliptic and glabrous; lower flower male or absent. Upper anthecium ovoid, glabrous, and indurate. Caryopsis ovoid; hilum punctiform, embryo ½ or more the length of the caryopsis.

*Etymology.* The name of the genus honors Dr. Jan F. Veldkamp, an outstanding agrostologist with very valuable publications in Paniceae of the Old World.

**Figure 2:**
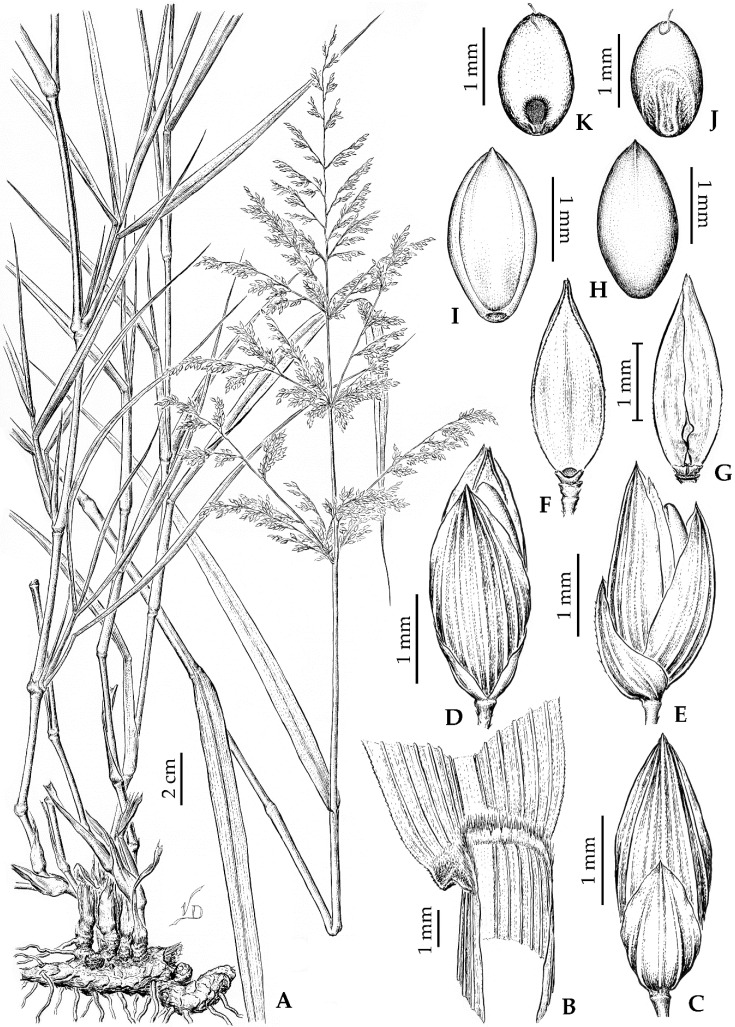
*Janochloa antidotale* (Retz.) Zuloaga & Delfini: (**A**). Plant; (**B**). Ligular region; (**C**). Spikelet, ventral view; (**D**). Spikelet, dorsal view; (**E**). Spikelet, lateral view; (**F**). Lower palea, dorsal view; (**G**). Lower palea, ventral view; (**H**). Upper anthecium, dorsal view; (**I**). Upper anthecium, ventral view; (**J**). Caryopsis, embryo view; (**K**). Caryopsis, hilum view. [(**A–K**): *F.O. Zuloaga* et al. *7091* (SI); Drawn by Vladimiro Dudás].

***Janochloa antidotale*** (Retz.) Zuloaga & Delfini, **comb. nov.** ≡ *Panicum antidotale* Retz. Observ. Bot. 4: 17. 1786 (1786–1787). TYPE: India. “Colitur in hortis Malabarorum”, desert regions, s.d., *J.G. Koenig s.n.* (lectotype, LD [barcode (bc)] 1218666!, designated as “holotype” by J.F. Veldkamp, Blumea 41(1): 187. 1996; isolectotypes, BM (bc) 000959558!, K (bc) 000674299!, possible isolectotypes, C (bc) 10017099!, C (bc) 10017100!). Figure 2.

*= Panicum miliare* Lam., Tabl. Encycl. 1: 173. 1791. TYPE: India. Without locality, s.d., *D. Sonnerat s.n.* (possible holotype, P-LAM!; isotypes, BAA (bc) 00002362! fragment ex P-LAM, US (bc) 00148304! fragment ex P-LAM).

*= Panicum proliferum* Lam., Tabl. Encycl. 4: 747. 1798. TYPE: Country and locality unknown, Hb. Lamarck (possible holotype, P-LAM!).

Plants perennial, with strong, long rhizomes, densely pilose cataphylls; culms 0.8–1.5 m tall and decumbent at the base, then erect, branching in the upper nodes; internodes 4–10 cm long, solid, rigid, glaucous, and glabrous; nodes brown and glabrous. Sheaths 4–7 cm long, as long as or shorter than the internodes; glabrous, the margins membranous. Ligules membranous-ciliate, 0.8–1.4 mm long, brownish, glabrous. Blades lanceolate, 18–38 × 0.8–1.5 cm, flat, glabrous, rounded at base, apex attenuate, the margins scabrous. Inflorescence terminal, pyramidal, exerted panicle, 14–25 × 5–20 cm, multi-flowered; main axis wavy, scabrous to smooth; pulvini shortly pilose; first order branches verticillate to alternate, spikelets solitary or paired and appressed on the branches; pedicels 7–3 mm long, claviform, scaberulous, with or without short, thick hairs. Spikelets long ovoid, 2.5–3.4 × 0.8–1.1 mm, gaping at maturity, glabrous, pale to brownish and tinged with purple. Glumes and lower lemma with manifest nerves and scabrous on the inner surface; lower glume ovate, ¾ the length of the spikelet, 3–5-nerved, acuminate, the margins hyaline; upper glume 2.1–3 mm long, shorter than the lower lemma, 5–9-nerved, acute; lower lemma 2.4–3.2 mm long, glumiform, 5–9-nerved, acute; lower palea elliptic, 2.2–3 × 0.7–1 mm, membranous, glabrous, the margins scaberulous; lower flower staminate or absent. Upper anthecium ovoid, 1.8–2.3 × 0.9–1.1 mm, glabrous, and pale, with dark spots at maturity, indurate, not stipitate, with simple papillae towards the apex of the palea. Caryopsis ovoid, 1.5–1.9 × 0.8–1.1 mm, pale; hilum punctiform, embryo ½ or more the length of the caryopsis.

*Common name.* “blue panic”, “giant panic”.

*Chromosome numbers. n* = 9 [64,65,66,67,68]; *n* = 14 [69]; 2*n* = 18 [67,70,71,72].

*Distribution and ecology.* Species native to India and widely distributed in Asia. [73] pointed out that it grows in dunes along rivers and in desert areas in general, where it survives drought seasons due to the strong rhizomes that the plants have; plants are bigger in more suitable regions. It is a valuable species for fixation of dunes and has low forage value. Introduced in Africa, Palestine, Australia, and America, from the United States, Mexico, and Mesoamerica to South America, from Colombia to Argentina.

*Additional specimens examined.* AFGHANISTAN. Jagdalek, 17 May 1936, *Koelz 8251* (US). ARGENTINA. Jujuy: Santa Bárbara, El Piquete, 24 October 1979, *Cabrera* et al. *31023* (SI, US); Camino de San Pedro a El Piquete, Ruta Prov. 1,6 km de El Piquete, 12 October 2000, *Zuloaga* et al. *7091* (SI); Ruta Prov. 1, El Piquete, 14 December 1986, *Zuloaga* et al. *2853* (SI). AUSTRALIA. Queensland: Biloela Research Station, 1 November 2004, *Dillon 5561* (BRI); Coolum, 20 January 1954, *Blake 19244* (BRI). Northern Territory: ca. 0.5 km NNW of Ghan Historical Reserve, Alice Springs, 25 March 1997, *Albrecht 8216* (NT). Western Australia: Carlton Beach Experimental Plots, Kununurra, 28 May 1944, *Gardner 7289* (CANB). BRAZIL. Rio de Janeiro: Universidade Rural, km 47, s.d., *Black 51-11421* (IAN, US). CHAD, Ferme de Deli près Moundou, 23 November 1962, *Gillet s.n.* (P (bc) 00957409). COLOMBIA. Atlántico: Mun. Puerto Colombia, en los alrededores del casco urbano, 3 July 1978, *de López 1000* (COL). Magdalena: Santa Marta, Parque Nacional Tayrona, Bahía Concha, February 1989, *Atehortúa 04* (SI); Parque Tayrona, Bahía Conchas, July 1986, *Nicora 8708* (SI); Mun. Santa Marta, centro de acopio “Prodeco, en valle arenoso, 4 October 1976, *Idrobo 8695* (COL); Isla de Salamanca, km 11, 25 June 1970, *de López 555* (COL). Tolima: Espinal, vivero del Servicio Técnico Agrícola Colombiano Americano, 8 January 1960, *Echeverry 127* (COL). Valle del Cauca: Mun. Palmira, Facultad de Agronomía de la Universidad Nacional de Colombia, 11 April 1962, *Idrobo 4993* (COL). ECUADOR. Manabí, 24 September 1984, *Laegaard 53065* (MO). INDIA. Punjab, 1 May 1933, *Koelz 4368* (US); Harayana, s.d., *Nair 21604* (MO); Pathankot, 7 May 1917, *Stewart & Stewart 1722* (NY). Without locality and collector, 1630 (K). ISRAEL. N Eilat, 12 April 1996, *Erikson s.n.* (OHN 112403). MEXICO. Baja California Norte: Seven miles NNW of Chapala on a rocky slope, 760 m, s.d., *Reeder & Reeder 7178* (US). Coahuila: Terrenos de la U.A.A.A.N., Buenavista, Saltillo, 24 September 1982, Villarreal 1772 (ANSM, MEXU). NEW CALEDONIA. Pres île de Népoui, 18 November 1987, *Veillon 6594* (P). NICARAGUA. Managua: Common in crops of Escuela Nacional de Agricultura y Ganadería de Nicaragua, La Calera, 22 January 1972, *Molina 27271* (F, MO, US); In the vicinity of Escuela Nacional de Agricultura y Ganadería, 12 km E of Managua, s.d., *Seymour 5446* (MO); 1 August 1972, *Seymour 6275a* (MO); 16 January 1969, *Seymour 2226* (MO); 25 July 1972, *Seymour 6094* (MO); at Escuela Agrícola, just E of Managua airport, along roadsides, 7 July 1970, *Pohl and Davidse 12218* (MO). PAKISTAN. Ca. 26 miles from Karachi on way to Bella, 16 August 1962, *Abedú 3662* (NY). UNITED STATES OF AMERICA. Arizona: Altar Valley, 16 km S of Robles Junction, 16 August 1990, *Reeder & Reeder 8546* (ANSM). Texas: 7 mi S of the junction of Hwy 16 and FM 2359 along HWY 16, 23 May 1986, *Hatch 5267* (ANSM); Keifer Ranch, 6 miles west of Batesville, 11 April 1981, *Freeman s.n.* (ANSM).

### 3.3. Setaria P. Beauv.

*Setaria* is currently recognized as a difficult and non-monophyletic genus with the species isolated or distributed in four blocks of clades or groups of clades within the Cenchrinae [15,29]. These clades are correlated and named according to their geographic origins (i.e., 1. Africa, tropical-Asia, 2. Australia, Australasia, 3. temperate Asia, and 4. the Americas) and none of the tested subgenera (i.e., *Ptychophyllum* and *Setaria*) were retrieved as monophyletic [15,29].

In our preliminary phylogeny, relationships among *Setaria* species were similar to those shown in previous *ndh*F-based phylogenies [15,29] with notable differences mainly in the composition of the proposed American clades. Here, most of the *Setaria* species from the Americas were resolved in two main clades, one major (clade B, corresponding to clade X of [29]) and one minor (clade E, corresponding to clade II of [29]), both morphologically quite distinct. The major clade was originally composed of South American perennial species; however, due to the placement of some taxa of the subgenera *Paurochaetium* and *Reverchoniae* [see Supporting Information Table S2] within this clade, its range was extended to Central and North America. Our findings also identified a clade related to the morphology of the species rather than its geographic origins (i.e., clade A), which groups *S. chapmanii*, a taxon previously treated in subgenus *Paurochaetium* [20] (Figure 1).

As expected, the subgenera *Paurochaetium* and *Reverchoniae* (Table S2) are non-monophyletic like the other subgenera of *Setaria*. Although they share morphological similarities, five of the six species analyzed (i.e., *S. distantiflora*, *S. leonis*, *S. reverchonii*, *S. variifolia*, and *S. utowanaea*) were resolved within the major American clade (clade B), according to their geographic origins. On the other hand, *S. chapmanii*, also an American species, was unambiguously strongly supported within clade A, related to other species with inflorescences “Paspalidium type”.

As mentioned earlier, the taxonomic history of subgenera *Paurochaetium* and *Reverchoniae* are linked to that of the genus *Paspalidium* [16,25,27,28,74]. Species of subgenus *Paurochaetium* were originally described as a subgenus of *Panicum* [75] to accommodate taxa in which setae are present only at the ends of the primary branches of the inflorescence. Subgenus *Paurochaetium* was first placed under *Setaria* at the rank of section by [76] and elevated to subgenus by [20]. Following [75]’s concept, [25] established the genus *Paspalidium*, segregating it from *Setaria*, but species of subgenus *Paurochaetium* were transferred to *Paspalidium* only decades later [74]. As the circumscriptions of the two genera overlap and the distinction between them is somewhat arbitrary, the *Paspalidium* species were transferred back to *Setaria* [16,27,28] (see Table S2 for a synopsis of species and different classifications for the taxa of subgenera *Paurochaetium* and *Reverchoniae*), a result supported by molecular analyses [1,15,29].

Subgenera *Paurochaetium* (five accepted species) and *Reverchoniae* (two accepted species) (Table S2) [18,26] include caespitose perennial plants distributed from the United States (New Mexico, West South Central, and Florida) to northern South America (Venezuela and Colombia), being mostly concentrated in the Caribbean [18,20,26,33,74]. *Setaria chapmanii*, analyzed here for the first time, grows on limestone, coral, shell or sandy soils in the Florida Keys, the Bahamas, Cuba, and the Yucatan Peninsula [20]; its panicles have branches with spikelets biseriate, the blunt first glume turned away from the rachis and the back of the upper lemma toward it, and a single bristle present below the terminal spikelets [74]. Although this species was previously treated within subgenus *Paurochaetium* [20], the well-ordered arrangement of its spikelets in unilateral spikes is highly anomalous in this group [74], as well as the lack of the lower palea [20]. In our phylogeny, *S. chapmanii* is placed in clade A and turned out to be the only species of subgenus *Paurochaetium* that is related to the others previously considered in *Paspalidium* (the remaining species of subgenus *Paurochaetium* were included in clade B).

Clade A groups species with inflorescences “Paspalidium type” related to wet/aquatic habitats and, except for *S. chapmanii*, which have slender culms, its members are characterized by having spongy culms [17,74,77]. The relationship between *S. chapmanii* and *S. geminata* for sharing the same type of habitat has been previously highlighted [74]. *Setaria geminata* is native to Africa and Asia, introduced unintentionally in tropical and subtropical areas of other continents [17]. It is an aquatic species with thick and spongy culms, while *S. chapmanii* inhabits temporary pools and marshes and is characterized by having culms mostly simple, erect, slender, and smooth [74,77]. The spongy culms of *S. geminata* are also shared with *S. punctata* and *S. uda*. From the former species, *S. geminata* is distinguished by having spikelets ovoid and lower palea well-developed while in *S. punctata* the spikelets are ellipsoid and lack lower palea [17]. *Setaria uda* is a species native to Australia and Papua New Guinea [17], and its position within clade A is confirmed here by the addition of a second voucher (i.e., *S. uda* 2). It differs from *S. geminata* and *S. punctata* mainly by having a caespitose habit; it lacks rhizomes and has smaller spikelets [17].

In [29], *S. magna*, *S. rara* and *Plagiosetum refractum* (F. Muell.) Benth. Were resolved as successive sisters to other “*Paspalidium*” species (i.e., *Setaria albovillosa* (S.T. Blake) R.D. Webster, *Setaria basiclada* (Hughes) R.D. Webster, *Setaria constricta* (Domin) R.D. Webster, *Setaria flavida* (Retz.) Veldkamp, *Setaria globoidea* (Domin) R.D. Webster, and *Setaria jubiflora* (Trin.) R.D. Webster); these relationships were not retrieved by our analyses with exception of *Plagiosetum refractum*, whose sister position to the “Australian Paspalidium clade” is poorly supported by BI and ML. *Setaria rara* is endemic to Australia, commonly found in arid areas associated with creeks or lagoons [17]. It was previously included in *Paspalidium* and shares an annual habit with *S. basiclada* [17]; however, the position of *S. rara* remains unresolved even with the addition of a second voucher. *Setaria magna* is also an annual species but is native to tropical and subtropical Americas and morphologically different from *Paspalidium*. It is distinguished from other *Setaria* species by its robust aspect with culms as much as 4 m tall and ligules forming an inverted “V” [18]. Although the placement of *S. magna* is not yet defined, our analyses corroborated that *S. magna* is not phylogenetically related to species of the American clades.

Clade B groups most of the American perennial species of *Setaria,* and as in previous studies [15,29], it was retrieved in all analyses. *Setaria cernua*, whose position was unclear in [29], was consistently supported within this clade, nested with *S. reverchonii*. *Setaria cernua* is characterized by having conspicuous superficial rhizomes, tillers with strongly keeled leaves resembling those of some Iridaceae, lower anthecium male with developed anthers, and upper anthecium shorter than the spikelet [18]. This unique combination of characters states led [18] to establish the monotypic subgenus *Cernatum* Pensiero, which was not supported by our findings, and disagrees with previous results [29] which had recovered it in an isolated position.

The two species of subgenus *Reverchoniae* (Table S2) were also placed in clade B, but they do not appear to be related to each other nor to the subgenus *Paurochaetium* taxa. Subgenus *Reverchoniae* was raised to accommodate species with panicles erect, spikelets randomly disposed on the branches, and the central inflorescence axis scabrous [26]. *Setaria variifolia* differs from *S. reverchonii* mainly in having the lower palea well-developed and by the geographic distribution in the Yucatán peninsula of Mexico and Mesoamerica (vs. lower palea absent or rudimentary and the geographically distributed in Texas, New Mexico, and Oklahoma (United States) and northern Mexico in *S. reverchonii*) [26]; the placement of *S. variifolia* within the larger American clade is confirmed here by sequencing of two vouchers but its relationships remain unknown.

*Setaria scheelei* has been assigned to subgenus *Setaria* [20] and was included in our analysis because it shares a geographic distribution pattern similar to that of subgenus *Paurochaetium* (i.e., native to southwest and south-central United States to Mexico). *Setaria scheelei* is a highly polymorphic species characterized by having a robust aspect; culms usually geniculate at the base, blades are usually flat and pubescent, and the upper lemma short-apiculate, incurved, and finely cross-wrinkled [20]. A sister relationship between *S. leonis* and *S. scheelei* was weakly supported in the Bayesian analysis but these two species are quite morphologically distinct and do not share ecological similarities. *Setaria leonis* has a more delicate aspect (30–60 cm tall), slender culms, erect or spreading, leaf blades broad but narrower than the sheath at the base, and a muticous upper lemma; it is commonly found in shad places on alluvial soils of limestone canyons and river bottoms while *S. scheelei* prefers open areas, rocky slopes, and clearings [20]. *Setaria leonis* shares the slender culms, geographic distribution, and habitat with *Setaria pradana* (León ex Hitchc.) León [20]; however, we were not able to analyze the latter species because the *ndh*F failed in all amplification attempts.

*Setaria hunzikeri*, here analyzed for the first time, was resolved as sister to one accession of *S. lachnea*, also within clade B. The two species are important forage grasses native in South America and are morphologically very similar [18], so their close relationship in our analyses is not surprising. *Setaria hunzikeri* differs from *S. lachnea* by having hirsute and narrower blades and smaller inflorescences up to 8 cm long (vs. glabrous or scabrous blades and inflorescences ranging from 7 to 25 cm long) [18].

*Setaria nicorae* was represented in [29]’s phylogeny by a partial sequence and placed in a polytomy together with other South American perennial species. Here, by including a second voucher with a complete *ndh*F sequence, we confirmed the placement of *S. nicorae* within the major American clade, but its relationships remain unknown. Morphological similarities between *S. nicorae* and *S. utowanaea* have been noted by [18], mainly because they share the caespitose habit with conspicuous rhizomes, ovoid spikelets, and 5–7-nerved upper glume; however, these two species are not phylogenetically related. *Setaria utowanaea* is sister to *S. distantiflora*, and both species are morphologically distinct but similar in general aspect and share the habitat (i.e., open areas and rocky soils). *Setaria distantiflora* is endemic to the Caribbean and is characterized by having a caespitose habit, lacking conspicuous rhizomes, ligules as a fringe of very short hairs, and lanceolate-ellipsoid spikelets while *S. utowanaea* has short rhizomes, membranous-ciliate ligules, ovoid spikelets and is more widely distributed (i.e., Caribbean, Colombia to Venezuela) [18,20].

As presented by [29], the minor American clade (clade E) groups annual species with “bottle-brush inflorescences” (i.e., cylindrical, dense, and continuous spiciform panicles), and both antrorse and retrorse prickles on the same bristle, the latter indicated as potential morphological synapomorphy of this clade [29]. In this phylogeny, bootstrap supports for relationships of clade E are weaker than that retrieved previously, possibly because of the placement of *S. longipila*, here analyzed for the first time, within this clade. *Setaria longipila* is also an annual species, but its subspiciform panicles [20] are distinctive. On the other hand, *Setaria grisebachii* E. Fourn., another annual species with inflorescences similar to those of *S. longipila*, is sister to *Setariopsis auriculata* (E. Fourn.) Scribn., and are both related to *Zuloagaea bulbosa* (Kunth) E. Bess. The position of *S. grisebachii* outside clade E will have to be verified by inclusion of multiple accessions and other morphologically similar American species (e.g., *Setaria liebmanni* E. Fourn.) in further analyses.

### 3.4. Cenchrus and Related Taxa

*Cenchrus* L. is a cosmopolitan genus with approximately 120 species [5] characterized by having one or several spikelets accompanied by one bristle or surrounded by an involucre of multiple bristles, or with bristles fused in a cup-like structure [13]. It is a monophyletic genus only when *Cenchropsis*, *Pennisetum*, *Kikuyuochloa*, the monotypic *Odontelytrum*, and *Snowdenia* are included within it [1,12,13,14]; however, recent molecular phylogenetic studies [3] and our findings showed *Cenchrus* paraphyletic with *Stereochlaena cameronii* embedded in the “Cenchrus clade”. *Stereochlaena cameronii* is morphologically quite distinct from *Cenchrus* in having digitate racemes, imbricate paired spikelets, and lower lemma awned [24]. Therefore, to reach any decision on the inclusion of this species within *Cenchrus*, its placement in the tree must be confirmed by additional accessions and more variable markers.

*Pseudochaetochloa australiensis* Hitchc., an endemic species to Australia, is considered as a synonym of *Cenchrus arnhemicus* (F. Muell.) Morrone in [33]; however, this treatment is not supported by our analyses. Here, *P. australiensis* formed a strongly supported clade with the dioecious Australian *Spinifex* and *Zygochloa*, which corroborates its classification as an independent genus of *Cenchrus*. *Pseudochaetochloa australiensis* is distinguished from these two by having monoecious two-flowered spikelets bearing a single bristle subtending many of the spikelets, lower anthecium well developed, and both lemmas membranous, similar in size, shape, and texture [78].

### 3.5. Stenotaphrum Trin.

*Stenotaphrum* is a primarily tropical genus including seven species [33,79,80] and, as in *Paspalidium*, its secondary-order inflorescence branches end in a bristle [80]. The placement of *Stenotaphrum* within subtribe Cenchrinae and its phylogenetic relationships have been uncertain due to limited data from previous studies (i.e., in [29] it was represented only by *Stenotaphrum secundatum* (Walter) Kuntze). Here, by increasing the number of species sampled (total of four), our results corroborated the close relationship of *Stenotaphrum* with *Setaria retiglumis* (syn. *Paspalidium retiglume* (Domin) Hughes) and *Uranthoecium truncatum*, retrieving it as a paraphyletic genus, as suggested in [3]. *Stenotaphrum* is distinguished from *Setaria* and the monotypic *Uranthoecium* by having the main inflorescences axis thickened and flattened, with the secondary branches embedded in it [79,80]. *Uranthoecium truncatum* is characterized by having short lateral branches, disarticulating rachis and truncate glumes, a set of features unique in this clade. As in previous analyses [29], here *U. truncatum* was also strongly (BI and MP) supported as sister to *S. retiglumis*. Both are caespitose annual species endemic to Australia and exhibit a very similar foliar anatomy [1,31], although *S. retiglumis* resembles other *Setaria* species in the phenotype.

## 4. Materials and Methods

### 4.1. Molecular Studies

#### 4.1.1. Taxon Sampling

The aligned data matrix used in the phylogenetic analyses includes a total of 180 accessions, of which 172 are ingroup corresponding to 22 (of 24) genera of the subtribe Cenchrinae [see Supporting Information Table S1]. The chloroplast DNA (cpDNA) *ndh*F matrix previously published [29], excluding the outgroup, was completed with 63 new sequences. Of these, we have sequenced 20 new accessions corresponding to species of the subgenera *Paurochaetium* and *Reverchoniae* of *Setaria* (Table S1, indicated with *) including those without a defined placement in [29] (Table S1, indicated with **) plus a second accession of *Alexfloydia repens* and *Panicum antidotale*. Eight species belonging to six closely related genera were selected as outgroup, based on [1,29]: *Aakia* (Paspaleae, Paspalinae), *Eriochloa* Kunth, *Moorochloa* Veldkamp, *Rupichloa* Salariato & Morrone, *Urochloa* P. Beauv. (Paniceae, Melinidinae) and *Panicum* (Paniceae, Panicinae). Information about vouchers and accession numbers of the new sequences obtained for this study and those available in GenBank are given in Table S1.

#### 4.1.2. DNA Amplification and Sequencing

Total genomic DNA was extracted from herbarium material using modified CTAB protocols from [81]. For the species that failed this protocol, the DNA was isolated using the DNeasy Plant Mini Kit (Qiagen, Hilden, Germany), following the manufacturer’s recommendations. Each species was amplified from a single voucher specimen, but a second voucher was also included for some taxa. The *ndhF* gene, coding NADH dehydrogenase subunit F, was amplified by polymerase chain reaction (PCR) and sequenced for each taxon. The complete region was amplified with a battery of primers in different combinations in four overlapping fragments using primer pairs specified by [39,82]: 5F–536R, 536F–972R, 972F–1666R, and 1666F–3R. Due to a lot of samples with a difficult amplification of the region 1666F–3R, a new reverse primer near the 3R region was designed for PCR amplification and sequencing of *ndh*F within the subfamily Panicoideae: 2150R (5′–TCTCCKATACAAAAACYARCAAKAC–3′).

PCR reactions were performed in a 25 µL final volume with 50–100 ng of template DNA, 5 µL Green Promega GoTaq^®^ buffer (5 u/µL), 0.5 µL MgCl_2_ (25 mM), 1.25 µL dNTP (10 mM), 1 µL of each primer (10 pM), and 0.3 µL of Taq polymerase (5 u/µL) provided by Promega (Madison, WI, USA). Variations in MgCl_2_ (0.5–1 µL) and total DNA dilutions (1:5, 1:10 and 1:50) were used. The reactions were carried out using the following parameters: one cycle of 95 °C for 2 min, 39 cycles of 95 °C for 30 s, 48 °C for 30 s, and 72 °C for 1.5 min, and a final extension cycle of 72 °C for 10 min. PCR products were run out on a 1% TBE (Tris-Borate-EDTA) agarose gel stained with SYBR Safe DNA gel stain (Invitrogen Life Technologies) and visualized in a blue-light transilluminator. Automated sequencing was performed by Macrogen, Inc. (Seoul, Republic of Korea). Forward and reverse strands were sequenced for all fragments, with a minimum overlap of 80%.

#### 4.1.3. Phylogenetic Analyses

Sequence editing and assembly were performed with MEGA v. 7.0 [83]. Accuracy of sequences was assessed by visual inspection of the chromatograms. Alignments were generated with Clustal X v. 2 [84] under the default settings and were trimmed to remove part of the 3′ end, for which many sequences were incomplete. The alignments obtained were then checked and improved manually, when necessary, by visual refinement using the program MEGA v. 7.0 [83]. The phylogenetic reconstruction was based on parsimony (MP) [85], maximum likelihood (ML) [86,87], and Bayesian inference (BI) [88] methods. In all analyses, gaps were considered as missing data. To determine the best-fitting nucleotide substitution model, data were submitted to jModeltest 2.1.1 [89] and the Akaike information criterion (AIC) selected TVM+I+G.

Parsimony analyses were performed using TNT ver. 1.1 [90] with Fitch parsimony [85] as the optimality criterion. All characters were equally weighted and treated as unordered. A heuristic search was conducted using 1000 random taxon-addition replicates, with the tree-bisection-reconnection (TBR) algorithm, saving up to 15 trees per replicate to prevent extensive swapping on islands with many trees. The resulting trees were then used as starting trees for a second-round search using TBR branch swapping with an upper limit of 10,000 trees. Nonparametric bootstrap support (BS) was estimated using 10,000 pseudo-replicates, and the same parameters were used in our MP analyses [91]. Bootstrap percentages of 50 to 80 were considered weak, 81 to 90 moderate, and >90 strong.

ML analyses were conducted using RAxML-HPC2 on XSEDE (v. 8.2.12) [92] in the Cyberinfrastructure for Phylogenetic Research (CIPRES) Portal v. 3.3 [93]. For these analyses we used the implemented algorithm, which allows one to perform optimal tree searches and obtain bootstrap support [91] in one single analysis [94]. To this end, we performed 1000 bootstrap replicates with a subsequent search of the maximum likelihood tree, using the GTRGAMMA nucleotide substitution model [92], individual per-site substitution rates (-c), and default setting of likelihood acceptance (-e), 25 and 0.1, respectively. Bootstrap percentages of 50 to 80 were considered weak, 81 to 90 moderate, and > 90 strong.

Bayesian analyses were performing using MrBayes v. 3.2.7a [95] in the CIPRES Portal [93] with nst = 6 and rates = invgamma. The datasets were analyzed in two independent runs of 10 million generations, each with four Markov chains (one cold and three heated chains), sampling every 1000 generations. Convergence and effective sample size (ESS) of the runs were assessed in Tracer v.1.7 [96], checking that ESS > 200 for all parameters. After discarding the initial 2500 trees of each run as burn-in (25%), the remaining trees (15,002) were summarized in a Maximum Clade Credibility Tree (MCCT) including the posterior probabilities (BPP) as branch support estimates. The cutoff for strong support in the Bayesian analyses was 0.95 (roughly equal to *p* < 0.05) posterior probabilities and values below 0.8 were considered not supported.

### 4.2. Taxonomic Treatment

The taxonomic treatment was based on bibliographical research including original descriptions, as well as analyses of specimens housed in the following herbaria (acronyms according to [97]): ANSM, BRI, CANB, COL, F, IAN, MEXU, MO, NT, NY, ONH, P, SI, and US. We examined types in person and images available online at the JSTOR Global Plants website (http://plants.jstor.org, accessed on 10 October 2022) and/or at the websites of the aforementioned herbaria. The protologues of all taxa have been checked. For each name, the place of valid publication is given followed by the holotype or lectotype. Accepted names are in bold italic. Complete synonymy was provided and cited in chronological order.

## 5. Conclusions

This study allowed us to present the morphological features of 24 genera of subtribe Cenchrinae of the Paniceae, including the geographic distribution of taxa and a key to distinguish them. Among the results, the genus *Acritochaete* was excluded from the subtribe, new taxa, of subgenera *Paurochaetium* and *Reverchoniae* were analyzed within the polyphyletic genus *Setaria,* relationships of *Cenchrus* with other genera, such as *Stereochlaena* and *Pseudochaetochloa* are also discussed as well as comments on the genus *Stenotaphrum*. Finally, the new monotypic genus *Janochloa*, segregated from *Panicum*, is phylogenetically analyzed, described, and fully illustrated.

## Data Availability

The aligned data matrix and trees from the three methods of analysis are available at Repositorio Institucional CONICET Digital under the following link http://hdl.handle.net/11336/163438, accessed on 28 July 2022.

## Supplementary Materials

The following supporting information can be downloaded at: https://www.mdpi.com/article/10.3390/plants12040749/s1, Table S1: Taxa, voucher information, and GenBank accession numbers for *ndh*F sequences. Species of the subgenera *Paurochaetium* and *Reverchoniae* of *Setaria* P. Beauv. and sequences obtained for this study are indicated with an asterisk (*); species without a defined placement in [29] are indicated with double asterisk (**). Accepted names for *Setaria* species follow [17,18], and the remainder Cenchrinae taxa plus the outgroup follow [33]; herbarium acronyms follow [97]; Table S2: A synopsis of species and different classifications for the subgenera *Paurochaetium* and *Reverchoniae* of *Setaria* P. Beauv. since they were erected from *Panicum* L.
