# Supplementary material for: An Update of the Cenchrinae (Poaceae, Panicoideae, Paniceae) and a New Genus for the Subtribe to Clarify the Dubious Position of a Species of Panicum L
Source: Plants (Basel). 2023 Feb 7;12(4):749. doi: 10.3390/plants12040749 (PMC9966601; doi:10.3390/plants12040749)
Supplement: Supplementary file 1 [file plants-12-00749-s001.zip › plants-2147392-supplementary.pdf]

Table S1. Taxa, voucher information, and GenBank accession numbers for *ndh*F sequences. Species of the subgenera *Paurochaetium* and *Reverchoniae* of *Setaria* P. Beauv. and sequences obtained for this study are indicated with an asterisk (*); species without a defined placement in [29] are indicated with double asterisk (**). Accepted names for *Setaria* species follow [17,18], and the remainder Cenchrinae taxa plus the outgroup follow [33]; herbarium acronyms follow [97].

Ingroup taxa (Cenchrinae)	Voucher information (from this study and when available)	GenBank accession
Acritochaete volkensii Pilg.	Guinea, North Bioko, *M. Carvalho 3691* (G)	HE573491
Alexfloydia repens B.K. Simon (1)	Australia, *S.J. & T.R. Hodkinson 9391* (TCD)	HE573538
Alexfloydia repens (2)	Australia, Cordwells Creek, *A.G. Floyd 2165* (BRS)	OQ383283*
Cenchrus abyssinicus (Hack.) Morrone	Ethiopia, Shewa, *G. Aweke 2912* (MO)	EU741938
Cenchrus alopecuroides Thunb.		AY029672
Cenchrus americanus (L.) Morrone		AF499149
Cenchrus caliculatus Cav.		EF189886
Cenchrus caudatus (Schrad.) Kuntze	South Africa, Mpumalanga, *E.A. Kellogg 1123* (MO)	EU741936
Cenchrus ciliaris L. (1)		AY029625
Cenchrus ciliaris L. (2)	South Africa, Mpumalanga, *E.A. Kellogg 1125* (MO)	EU741937
Cenchrus echinatus L.		AF499151
Cenchrus flaccidus (Griseb.) Morrone		AF499150
Cenchrus longisetus M.C. Johnst.		EF189888
Cenchrus mutilatus Kuntze		AY188498
Cenchrus myosuroides Kunth		AF499152
Cenchrus pilosus Kunth		EF189887
Cenchrus setaceus (Forssk.) Morrone		AY029673
Cenchrus setiger Vahl		AF499153
Chamaeraphis hordeacea R. Br.		JN604680
Dissochondrus biflorus (Hildebr.) Kuntze		JN604686
Hygrochloa aquatica Lazarides		JN604690
Ixophorus unisetus (J. Presl) Schltdl. (1)		AY623749
Ixophorus unisetus (2)	Mexico, Michoacán, *O. Morrone 3638* (SI)	EU741940
Panicum antidotale Retz. (1) (=*Janochloa antidotale* (Retz) Zuloaga & Delfini)	Argentina, Jujuy, *F.O. Zuloaga 7091* (SI)	AY188456
Panicum antidotale (2)	Colombia, Magdalena, *L. Atehortúa 04* (SI)	OQ383284*
Paractaenum novae-hollandiae P. Beauv.		JN604697
Paratheria prostrata Griseb.		JN604699
Plagiosetum refractum (F. Muell.) Benth. (1)	Australia, locality not indicated, *S. Jacobs 9600* (NSW)	EU819409
Plagiosetum refractum (2)	Australia, locality not indicated, *S.J. & T.R. Hodkinson 9600* (TDC)	HE573564
Pseudochaetochloa australiensis Hitchc.		JN604702
Pseudoraphis paradoxa (R. Br.) Pilg.		EF189892
Pseudoraphis spinescens (R. Br.) Vickery		MF998495
Setaria albovillosa (S.T. Blake) R.D. Webster	Australia, Queensland, *Clarkson & Henderson 7926* (CANB)	EU741984
Setaria alonsoi Pensiero & Anton	Argentina, Córdoba, *J.F. Pensiero 6973* (SF)	EU741943
Setaria appendiculata (Hack.) Stapf	Namibia, locality not indicated, *Giess 13524* (WIND)	EU747685

Setaria atrata Hack. (1)	Ethiopia, Wollega, *Gilbert & Thulin 729* (MO)	EU747689
Setaria atrata (2)	Madagascar, locality not indicated, *RGD 196* (K)	MF998497
Setaria barbata (Lam.) Kunth (1)		AF499145
Setaria barbata (2)	Venezuela, Distrito Federal, *O. Morrone 4804* (SI)	EU741944
Setaria basiclada (Hughes) R.D. Webster	Australia, South Australia, *Bates 46269* (AD)	EU741978
Setaria cernua Kunth (1)**	Ecuador, Pichincha, *S. Lægaard 70467* (MO)	EU741945
Setaria cernua (2)	Ecuador, Pichincha, *I. Grignon 84160* (MO)	OQ383285*
Setaria chapmanii (Vasey) Pilg.*	Mexico, Quintana Roo, *G. Davidse 20121* (MO)	OQ383286*
Setaria constricta (Domin) R.D. Webster	Australia, Western Australia, *Lepschi & Lally 2643 (*AD)	EU741979
Setaria distantiflora (A. Rich.) Pilg.*	Cuba, Mariel, *E.L. Ekman 1032* (MO)	OQ383287*
Setaria faberi R.A.W. Herrm.	China, Xiushiu, *Liu 890105* (MO)	EU741946
Setaria fiebrigii R.A.W. Herrm. (1)	Argentina, Entre Ríos, *F.O. Zuloaga 7044* (SI)	EU741947
Setaria fiebrigii (2)	Argentina, Misiones, *F.O. Zuloaga 7134* (SI)	EU741948
Setaria flavida (Retz.) Veldkamp (1)		EF189889
Setaria flavida (2)		EF189890
Setaria flavida (3)	Australia, New South Wales, *Moore 7584* (CANB)	EU741980
Setaria geminata (Forssk.) Veldkamp (1)**		AY029662
Setaria geminata (2)	Argentina, Corrientes, *S.S. Aliscioni 718* (SI)	OQ383288*
Setaria globoidea (Domin) R.D. Webster	Australia, New South Wales, *Lloyd 1007* (CANB)	EU741981
Setaria globulifera (Steud.) Griseb.	Argentina, Entre Ríos, *F.O. Zuloaga 7041* (SI)	EU741949
Setaria grandis Stapf	Malawi, Chelidini Valley, *Phillips 1070* (MO)	EU747688
Setaria grisebachii E. Fourn.		AF499141
Setaria hassleri Hack.	Argentina, Misiones, *S.S. Aliscioni 533* (SI)	EU741950
Setaria homonyma (Steud.) Chiov.	South Africa, KwaZulu-Natal, *E.A. Kellogg 1113* (MO)	EU741988
Setaria hunzikeri Anton	Argentina, San Luis, *L. Corradi 119* (BAA)	OQ383289*
Setaria italica (L.) P. Beauv.		AF499140
Setaria jubiflora (Trin.) R.D. Webster		EF189891
Setaria kagerensis Mez (1)	Nigeria, Jos Plateau, *Tuley 1579* (K)	EU741951
Setaria kagerensis (2)	Angola, Maiombe, *s.c. 9021* (US)	EU741971
Setaria lachnea (Nees) Kunth (1)	Argentina, Córdoba, *L.M. Giussani* 327 (SI)	EU741952
Setaria lachnea (2)	Argentina, Misiones, *F.O. Zuloaga 6758* (SI)	EU741953
Setaria leonis (Ekman ex Hitchc.) León*	Turks & Caicos, South Caicos, *D.L. Correll 49273* (MO)	OQ383290*
Setaria leucopila (Scribn. & Merr.) K. Schum.	Argentina, Salta, *M.A. Beilstein 03-127* (MO)	EU741954
Setaria lindenbergiana (Nees) Stapf	South Africa, Gauteng, *E.A. Kellogg 1136* (MO)	EU741994
Setaria longipila E. Fourn. (1)	Mexico, Sierra Madre, *Rose 2017* (US)	OQ383291*
Setaria longipila (2)	Honduras, Depto. Morazán, *Molina 3204* (US)	OQ383292*
Setaria longiseta P. Beauv.	Tanzania, Kigoma, *R.E. Gereau 6013* (MO)	EU741955
Setaria macrostachya Kunth (1)	Argentina, Córdoba, *J.F. Pensiero 6961* (SF)	EU747691
Setaria macrostachya (2)	Argentina, Salta, *O. Morrone 4638* (SI)	EU741956
Setaria madecassa A. Camus	Madagascar, locality not indicated, *M.S. Vorontsova 979* (K)	LN908172
Setaria magna Griseb. (1)**	Argentina, Corrientes, *S.S. Aliscioni 577* (SI)	EU741957
Setaria magna (2)	Argentina, Corrientes, *S.S. Aliscioni 707* (SI)	OQ383293*
Setaria mendocina Phil.	Argentina, San Luis, *F.O. Zuloaga 8085* (SI)	EU741958
Setaria nicorae Pensiero (1)	Argentina, Formosa, *J.F. Pensiero 6909* (SI)	EU747686
Setaria nicorae (2)	Argentina, Chaco, *S.S. Aliscioni 644* (SI)	OQ383294*

Setaria nigrirostris (Nees) T. Durand & Schinz (1)	South Africa, Gauteng, *E.A. Kellogg 1140* (MO)	EU741991
Setaria nigrirostris (2)	South Africa, Limpopo, *E.A. Kellogg 1133* (MO)	EU741992
Setaria nigrirostris (3)	South Africa, Limpopo, *E.A. Kellogg 1132* (MO)	EU742004
Setaria nigrirostris (4)	South Africa, KwaZulu-Natal, *E.A. Kellogg 1114* (MO)	EU741989
Setaria nigrirostris (5)	Tanzania, Iringa, *Greenway & Kanuri 14010* (MO)	EU741941
Setaria nigrirostris (6)	South Africa, KwaZulu-Natal, *E.A. Kellogg 1120* (MO)	EU741990
Setaria nigrirostris (7)	Madagascar, locality not indicated, *M.S. Vorontsova 1641* (K)	LN908171
Setaria oblongata (Griseb.) Parodi	Argentina, Córdoba, *J.F. Pensiero 6972* (SI)	EU741959
Setaria orthosticha R.A.W. Herrm.	Uganda, Kachwekano, *Purseglove 3331* (K)	EU747690
Setaria palmifolia (J. Koenig) Stapf (1)		AF499144
Setaria palmifolia (2)		AY029680
Setaria pampeana Parodi ex Nicora (1)	Argentina, Salta, *A.M. Cialdella 230* (SI)	EU741961
Setaria pampeana (2)	Argentina, Córdoba, *L.M. Giussani 325* (SI)	EU741960
Setaria parviflora (Poir.) Kerguélen (1)		AF499143
Setaria parviflora (2)		AF499142
Setaria parviflora (3)		AY029678
Setaria parviflora (4)	Argentina, Entre Ríos, *E.R. Guaglianone 3257* (SI)	EU742003
Setaria parviflora (5)	Argentina, Entre Ríos, *E.R. Guaglianone 3258* (SI)	EU742000
Setaria paucifolia (Morong) Lindm.	Argentina, Corrientes, *S.S. Aliscioni 516* (SI)	EU741962
Setaria perrieri A. Camus	Madagascar, locality not indicated, *M.S. Vorontsova 1414* (K)	LN908170
Setaria petiolata Stapf & C.E. Hubb.	Madagascar, locality not indicated, *Grosvenor & Renz 1295* (K)	EU741963
Setaria pflanzii Pensiero	Argentina, Jujuy, *J.F. Pensiero 7056* (SI)	EU741964
Setaria plicata (Lam.) T. Cooke	Papua New Guinea, Bougainville, *Waterhouse 6145* (CANB)	EU741965
Setaria pumila (Poir.) Roem. & Schult.		EF189894
Setaria punctata (Burm. f.) Veldkamp	Locality not indicated, *M. Norsangsri 2387* (KKU)	HE575807
Setaria rara (R. Br.) R.D. Webster (1)**	Australia, Queensland, *Bean 4818* (CANB)	EU741982
Setaria rara (2)	Australia, locality not indicated, *M. Lazarides 8298* (AD)	OQ383295*
Setaria restioidea (Franch.) Stapf	Central Africa, Bamingui-Bangoran, *J.M. Fay 6007* (MO)	EU741967
Setaria retiglumis (Domin) R.D. Webster	Australia, Darwin, *Dunlop 3514* (CANB)	EU741983
Setaria reverchonii (Vasey) Pilg.*	United States, Texas, *Colquitt 25* (US)	OQ383296*
Setaria rosengurttii Nicora	Argentina, Entre Ríos, *O. Morrone 5211* (SI)	EU741968
Setaria sagittifolia (A. Rich.) Walp.	Madagascar, locality not indicated, *NOP 207* (K)	MF998496
Setaria scabrifolia (Nees) Kunth	Brazil, Paraná, *H.M. Longhi-Wagner 9464* (SI)	EU741969
Setaria scandens Schrad.	Bolivia, Santa Cruz, *O. Morrone 5077* (SI)	EU741970
Setaria scheelei (Steud.) Hitchc.*	United States, Texas, *J.R. Swallen 1580* (US)	OQ383297*
Setaria scottii (Hack.) A. Camus	Madagascar, locality not indicated, *M.S. Vorontsova 1041* (K)	MF998501
Setaria sphacelata (Schumach.) Stapf & C.E. Hubb. ex Moss (1)		EF189893
Setaria sphacelata (2)	South Africa, Mpumalanga, *E.A. Kellogg 1126* (MO)	EU741986
Setaria sphacelata (3)	South Africa, KwaZulu-Natal, *E.A. Kellogg 1143* (MO)	EU742002
Setaria sphacelata (4)	Argentina, Misiones, *F.O. Zuloaga 7222* (SI)	EU741972
Setaria sphacelata (5)	South Africa, Mpumalanga, *E.A. Kellogg 1121* (MO)	EU741997
Setaria sphacelata (6)	South Africa, Eastern Cape, *E.A. Kellogg 1104* (MO)	EU742001
Setaria sulcata (Schult.) Kunth (1)	South Africa, Mpumalanga, *E.A. Kellogg 1122* (MO)	EU741987

Setaria sulcata (2)	South Africa, KwaZulu-Natal, *E.A. Kellogg 1110* (MO)	EU741995
Setaria sulcata (3)	South Africa, KwaZulu-Natal, *E.A. Kellogg 1112* (MO)	EU741996
Setaria sulcata (4)	Australia, New South Wales, *A. Doust 1368* (MO)	AF499146
Setaria sulcata (5)	Australia, New South Wales, *A. Doust 1369* (MO)	AF499147
Setaria sulcata (6)	Locality not indicated, *H. Schaefer 2008/378* (BM)	HE575758
Setaria sulcata (7)	Madagascar, locality not indicated, *M.S. Vorontsova 310* (K)	LN908173
Setaria tenacissima Schrad.	Bolivia, La Paz, *O. Morrone 4860* (SI)	EU741973
Setaria uda (S.T. Blake) R.D. Webster (1)**	Australia, North Kennedy, *M. Lazarides 8165* (CANB)	EU747687
Setaria uda (2)	Australia, locality not indicated, *Cowie & Dunlop 7888* (CANB)	OQ383298*
Setaria utowanaea (Scribn.) Pilg. (1)*	Colombia, La Guajira, *P. Bunch 181* (MO)	OQ383299*
Setaria utowanaea (2)	Venezuela, Eduardo Miranda, *J.A. Steyermark 102307* (MO)	OQ383300*
Setaria vaginata Spreng.	Argentina, Misiones, *F.O. Zuloaga 7124* (SI)	EU741974
Setaria variifolia (Swallen) Davidse (1)*	Mexico, Campeche, *E.M. Martínez Salas 27648* (MO)	OQ383301*
Setaria variifolia (2)	Mexico, Campeche, *E.M. Martínez Salas 27089* (MO)	OQ383302*
Setaria vatkeana K. Schum.	Madagascar, locality not indicated, *M.S. Vorontsova 1813* (K)	MF998503
Setaria verticillata (L.) P. Beauv. (1)		EF189900
Setaria verticillata (2)	South Africa, Gauteng, *E.A. Kellogg 1138* (MO)	EU741985
Setaria verticillata (3)		AF499139
Setaria verticillata (4)	Argentina, Santiago del Estero, *J.F. Pensiero 7082* (SF)	EU741942
Setaria verticilliformis Dumort.	Australia, South Australia, *Alcock 192* (AD)	EU741975
Setaria viridis (L.) P. Beauv.		U21976
Setaria vulpiseta (Lam.) Roem. & Schult. (1)	Bolivia, Santa Cruz, *O. Morrone 5023* (SI)	EU741977
Setaria vulpiseta (2)	Paraguay, Amambay, *F.O. Zuloaga 7293* (SI)	EU741976
Setaria sp. 1	South Africa, Limpopo, *E.A. Kellogg 1129* (MO)	EU741998
Setaria sp. 2	South Africa, Limpopo, *E.A. Kellogg 1130* (MO)	EU741999
Setariopsis auriculata (E. Fourn.) Scribn.		JN604705
Spinifex littoreus (Burm. f.) Merr.	Australia, Perth, *E.A. Kellogg 1021* (MO)	EU741993
Spinifex sericeus R. Br.		EF189895
Stenotaphrum dimidiatum (L.) Brongn. (1)	Locality not indicated, *P. Rondeau 04-2005* (G)	AM849189
Stenotaphrum dimidiatum (2)	Madagascar, locality not indicated, *M.S. Vorontsova 632* (K)	MF998499
Stenotaphrum oostachyum Baker	Madagascar, locality not indicated, *M.S. Vorontsova 1042* (K)	LN908182
Stenotaphrum secundatum (Walter) Kuntze		AY029684
Stenotaphrum unilaterale Baker	Madagascar, locality not indicated, *M.S. Vorontsova 1043* (K)	MF998500
Stereochlaena cameronii (Stapf) Pilg.		JN604708
Uranthoecium truncatum (Maiden & Betche) Stapf (1)	Australia, locality not indicated, *S. Jacobs 9599* (NSW)	EU819410
Uranthoecium truncatum (2)	Australia, locality not indicated, *S.J. & T.R. Hodkinson 9604* (TCD)	HE573565
Whiteochloa airoides (R. Br.) Lazarides	Australia, Northern Territory, *A.P. Roberts 4023* (NT)	MG581794
Whiteochloa biciliata Lazarides	Australia, Western Australia, *A.A. Mitchell 7974* (NT)	MG581799
Whiteochloa capillipes (Benth.) Lazarides (1)		JN604714
Whiteochloa capillipes (2)	Australia, Northern Territory, *L.G. Adams 3046* (NSW)	MG581795
Whiteochloa cymbiformis (Hughes) B.K. Simon	Australia, locality not indicated, *Glober 20* (CANB)	MG581797
Whiteochloa multiciliata Lazarides	Australia, Northern Territory, *M. Lazarides 100* (AD)	MG581798

Whiteochloa semitonsa (F. Muell. ex Benth.) C.E. Hubb.	Australia, Northern Territory, *A.A. Mitchell 15642* (NT)	MG581796
Xerochloa barbata R. Br.	Australia, locality not indicated, *S.J. & T.R. Hodkinson 9323* (TCD)	HE573526
Xerochloa laniflora Benth.		JN604715
Zuloagaea bulbosa (Kunth) E. Bess (1)	United States, New Mexico, *E. Bess 41* (MO)	AY864833
Zuloagaea bulbosa (2)		AY029648
Zygochloa paradoxa (R. Br.) S.T. Blake (female)		EF189897
Zygochloa paradoxa (male)		EF189896
**Outgroup taxa**		
Aakia tuerckheimii (Hack.) J.R. Grande	Mexico, Chiapas, *A.L. Cabrera 12312* (SI)	KF982003
Eriochloa acuminata (J. Presl) Kunth	Mexico, Coahuila, *F.O. Zuloaga 9737* (SI)	GU594634
Eriochloa punctata (L.) Desv. ex Ham.	Argentina, Misiones, *F.O. Zuloaga 6838* (SI)	FJ486528
Moorochloa eruciformis (Sm.) Veldkamp	Argentina, Entre Ríos, *F.O. Zuloaga 7045* (SI)	AY188452
Panicum miliaceum L.		AY188472
Rupichloa acuminata (Renvoize) Salariato & Morrone	Brazil, Bahia, *F.O. Zuloaga 9029* (SI)	AY029692
Urochloa deflexa (Schumach.) H. Scholz	Locality not indicated, *G. Besnard 47-2006* (G)	AM849200
Urochloa panicoides P. Beauv.	Mexico, Tejupilco, *F.O. Zuloaga 7358* (SI)	FJ486519

Table S2. A synopsis of species and different classifications for the subgenera *Paurochaetium* and *Reverchoniae* of *Setaria* P. Beauv. since they were erected from *Panicum* L.

[75]	[20]	[74]	[18]	[26]		Accepted names according to [33]
Panicum subg. *Paurochaetium* Hitchc. & Chase	*Setaria* subg. *Paurochaetium* (Hitchc. & Chase) Rominger	*Paspalidium* Stapf	*Setaria* subg. *Setaria*	*Setaria* subg. *Paurochaetium* (Hitchc. & Chase) Rominger	*Setaria* subg. *Reverchoniae* W.E. Fox	
Panicum chapmanii Vasey	*Setaria chapmanii* (Vasey) Pilg.	*Paspalidium chapmanii* (Vasey) R.W. Pohl	*Paspalidium chapmanii* (Vasey) R.W. Pohl	*Setaria chapmanii* (Vasey) Pilg.		*Setaria chapmanii* (Vasey) Pilg.
Panicum distantiflorum A. Rich.	*Setaria distantiflora* (A. Rich.) Pilg.	*Paspalidium distantiflorum* (A. Rich.) Davidse & R.W. Pohl	*Setaria distantiflora* (A. Rich.) Pilg.	*Setaria distantiflora* (A. Rich.) Pilg.		*Setaria distantiflora* (A. Rich.) Pilg.
Panicum leonis Ekman ex Hitchc.	*Setaria leonis* (Ekman ex Hitchc.) León	*Paspalidium leonis* (Ekman ex Hitchc.) Davidse & R.W. Pohl	*Setaria leonis* (Ekman ex Hitchc.) León	*Setaria leonis* (Ekman ex Hitchc.) León		*Setaria leonis* (Ekman ex Hitchc.) León
Panicum pradanum León ex Hitchc.	*Setaria pradana* (León ex Hitchc.) León	*Paspalidium pradanum* (León ex Hitchc.) Davidse & R.W. Pohl	*Setaria pradana* (León ex Hitchc.) León	*Setaria pradana* (León ex Hitchc.) León		*Setaria pradana* (León ex C.L. Hitchc.) León
Panicum utowanaeum Scribn.	*Setaria utowanaea* (Scribn.) Pilg.	*Paspalidium utowanaenum* (Scribn.) Davidse & R.W. Pohl	*Setaria utowanaea* (Scribn.) Pilg.	*Setaria utowanaea* var. *utowanaea* (Scribn.) Pilg.		*Setaria utowanaea* (Scribn.) Pilg.
Panicum ophiticola Hitchc. & Ekman	*Setaria ophiticola* (Hitchc. & Ekman) León	*Paspalidium ophiticola* (Hitchc. & Ekman) Davidse & R.W. Pohl	*Setaria ophiticola* (Hitchc. & Ekman) León	*Setaria utowanaea* var. *ophiticola* (Hitchc. & Ekman) W.E. Fox		*Setaria utowanaea* (Hitchc. & Ekman) W.E. Fox.
	Setaria subtransiens Hitchc. & Ekman	*Paspalidium subtransiens* (Hitchc. & Ekman)	*Setaria subtransiens* Hitchc. & Ekman	*Setaria utowanaea* var. *subtransiens* (Hitchc. & Ekman) W.E. Fox		*Setaria utowanaea* (Hitchc. & Ekman) W.E. Fox

		Davidse & R.W. Pohl			
Panicum reverchonii Vasey	*Setaria reverchonii* (Vasey) Pilg.		*Setaria reverchonii* (Vasey) Pilg.	*Setaria reverchonii* subsp. *reverchonii* Vasey) Pilg.	*Setaria reverchonii* subsp. *reverchonii* (Vasey) Pilg.
Panicum firmulum Hitchc. & Chase	*Setaria firmula* (Hitchc. & Chase) Pilg.		*Setaria firmula* (Hitchc. & Chase) Pilg.	*Setaria reverchonii* subsp. *firmula* (Hitchc. & Chase) W.E. Fox	*Setaria reverchonii* subsp. *firmula* (Hitchc. & Chase) W.E. Fox
Panicum ramisetum Scribn.	*Setaria ramiseta* (Scribn.) Pilg.		*Setaria ramiseta* (Scribn.) Pilg.	*Setaria reverchonii* subsp. *ramiseta* (Scribn.) W.E. Fox	*Setaria reverchonii* subsp. *ramiseta* (Scribn.) W.E. Fox
				Setaria variifolia (Swallen) Davidse	*Setaria variifolia* (Swallen) Davidse
